# *In vitro* gill cell monolayer successfully reproduces *in vivo* Atlantic salmon host responses to *Neoparamoeba perurans* infection

**DOI:** 10.1016/j.fsi.2018.11.029

**Published:** 2019-03

**Authors:** Irene Cano, Nick GH. Taylor, Amanda Bayley, Susie Gunning, Robin McCullough, Kelly Bateman, Barbara F. Nowak, Richard K. Paley

**Affiliations:** a Centre for Environment, Fisheries and Aquaculture Science, Barrack Road, The Nothe, Weymouth, Dorset, DT4 8UB, United Kingdom; b IMAS, University of Tasmania, Locked Bag 1370, Launceston, 7250, Tasmania, Australia

**Keywords:** Amoebic gill disease, AGD, *Neoparamoeba perurans*, RTgill-W1, Transwell^®^ permeable supports, AG-2, Th2, Phagocytosis

## Abstract

An *in vitro* model to study the host response to *Neoparamoeba perurans*, the causative agent of amoebic gill disease (AGD), was evaluated. The rainbow trout gill derived cell line, RTgill-W1, was seeded onto permeable cell culture supports and maintained asymmetrically with apical seawater. Cells were inoculated with either a passage attenuated or a recent wild clone of *N. perurans*. Amoebae, loaded with phagocytosed fluorescent beads, were observed associated with host cells within 20 min post inoculation (pi). By 6 h small foci of cytopathic effect appeared and at 72 h cytolysis was observed, with total disruption of the cell monolayer at 96 h pi. Due to cell monolayer disruption, the platform could not support proliferation of amoebae, which showed a 3-log reduction in parasite 18S rRNA mRNA after 72 h (10^6^ copies at 1 h to 10^3^ at 72 h pi). SEM observations showed amoebae-like cells with either short pseudopodia and a malleiform shape, or, long pseudopodia embedded within the gill cells and erosion of the cell monolayer. To study the host immune response, inoculated gill cells were harvested from triplicate inserts at 0, 1, 3, 6, 24 and 48 h pi, and expression of 12 genes involved in the Atlantic salmon response to AGD was compared between infected and uninfected cells and between amoebic clones. Both clones induced similar host inmate immune responses, with the up-regulation of proinflammatory cytokine IL1β, complement C3 and cell receptor MHC-1. The Th2 pathway was up-regulated, with increased gene expression of the transcription factor GATA3, and Th2 cytokines IL10, IL6 and IL4/13A. PCNA and AG-2 were also up-regulated. The wild clone induced significantly higher up-regulation of IL1β, MHC-1, PCNA, lysozyme and IL10 than the attenuated clone for at least some exposure times, but AG-2 gene expression was higher in cells inoculated with the attenuated one. A principal component analysis showed that AG-2 and IL10 were key genes in the *in vitro* host response to *N. perurans*. This *in vitro* model has proved to be a promising tool to study host responses to amoebae and may therefore reduce the requirement for in *vivo* studies when evaluating alternative therapeutants to AGD control.

## Highlights

• *In vitro* gill epithelia model enabled study of host response to *Neoparamoeba perurans*.
• The association of the parasite to the monolayer was studied by SEM.
• A protocol for live cell imaging of *N. perurans* is described.
• Host cytokines related to Th2 and cell proliferative pathways were up-regulated.

## 1 Introduction

Amoebic gill disease (AGD) is a serious disease affecting Atlantic salmon, *Salmo salar* (Linnaeus, 1758). and coho salmon, *Oncorhynchus kisutch* Karuk (Walbaum, 1792) farmed in the marine environment [1]. First reported in Tasmania Australia and Washington State and California USA in 1988 [2], AGD has since become endemic in Tasmania [3], and has subsequently impacted salmonid production in Scotland, France, Spain, Ireland, Norway, Chile, Canada, South Africa, Korea and Faroe Islands [1,[4], [5], [6], [7], [8]]. In addition to causing disease in salmonids, AGD has been reported in turbot *Scophthalmus maximus* L., ayu *Plecoglossus altivelis* (Temminck & Schlegel, 1846) and ballan wrasse *Labrus bergytta* (Ascanius, 1767) [see Refs. [[9], [10], [11]]].

The causative agent of AGD is the protozoan *Neoparamoeba perurans* [see Refs. [12,13]], which is the most phylogenetically divergent *Neoparamoeba* species [14]. Though normally free living, *N. perurans* can colonise the gills and cause the disease, which is characterised by multifocal white patches on the gill surface. At a histological level AGD causes hyperplasia of the epithelial and mucous cells, which can lead to lamellar fusion, generally in association with attached amoebae [15]. Cumulative mortalities can reach up to 50% if left untreated [16].

Currently, a commercial AGD vaccine is not available [17]. Though preliminary studies have been conducted to evaluate the efficacy of several potential chemotherapeutants [[18], [19], [20]], at present, exposure to freshwater remains the most effective treatment [21]. One of the key challenges to developing and evaluating new therapeutants is the availability of a cost effective ethically sound model system. *In vitro* systems have the potential to address these requirements, and, due to their clonal nature demonstrate less inherent heterogeneity between replicates than would be observed between live fish replicates, thus potentially reducing the need for animal use in experiments [22].

An *in vitro* system to study host-pathogen interaction in AGD requires the ability to isolate and grow the parasite and the ability to maintain suitable host cells. Protocols for the isolation of *N. perurans* from diseased fish, and culture onto malt yeast agar (MYA) are available. Under these conditions, the parasite retains its virulence and capacity to cause AGD in Atlantic salmon after at least 70 days of clonal culture [12]. However, cultured *N. perurans* has been shown to lose virulence after 3 years of repeated passage in *in vitro* culture [23].

There is little published information about *N. perurans* infection *in vitro*. A closely related parasite *N. pemaquidensis*, also isolated from AGD-affected fish [24], has been studied on an epithelial cell line derived from rainbow trout, *Oncorhynchus mykiss* (Walbaum, 1792) gills (RTgill-W1) [25]. When cultured at an osmolarity above 700 mOsm kg^−1^, this system has been shown to support the growth of *N. pemaquidensis* [see Ref. [27]]. However, unlike *N. pemaquidensis, N. perurans* requires full salinity sea water and cannot be exposed to host cells in cell culture media which have lower osmolality than sea water [27]. Transwell^®^ culture inserts provide a permeable support on which seeded cells can attach and form confluent monolayers. By replacing apical media with either freshwater or seawater, culture conditions can be modified to establish asymmetrical systems which produce a cell culture environment that enables the establishment of effective polarised epithelia and more closely resembles the *in vivo* state. This system has been used effectively to undertake chemotaxis assays, drug transport, and toxicity tests with fish gill primary cell cultures [see 29 for review]. RTgill-W1 cells can grow on a Transwell^®^ in direct contact with fresh or saltwater on their apical surfaces forming tight epithelia, and have been proposed as a sentinel model for *in vitro* aquatic toxicology [29], allowing the study of gill diseases and may therefore be suited to studies on *N. perurans*.

The aim of this study is to test an *in vitro* platform as a model to study host-*N. perurans* interactions, by using the rainbow trout gill cell line RTgill-W1 seeded onto Transwell^®^ inserts and exposed to two *N. perurans* clones: a wild type clone and a laboratory passage attenuated one. The *in vitro* association of *N. perurans* with the gill epithelium, the parasite growth and the expression of a selection of genes involved in the Atlantic salmon innate immune response to AGD are analysed. The potential application of this platform as an *in vitro* proxy to evaluate therapeutics to combat AGD is discussed.

## 2 Material & methods

### 2.1 Ethics statement

Animal procedures were approved by the Animal Welfare and Ethical Review Body (AWERB) at the Cefas Weymouth Laboratory and conducted in compliance with the Animals (Scientific Procedures) Act 1986.

### 2.2 *Neoparamoeba perurans* isolates and culture

*N. perurans* trophozoites were isolated from the gills of naturally infected Scottish farmed sea-cage Atlantic salmon showing typical AGD lesions as described before [30]. Isolated amoebae were then cultured on malt yeast agar (MYA: 0.01% malt, 0.01% yeast, 2% Bacto agar, 0.2 μm filtered sea water (SW) at 35‰ salinity) overlaid with 0.2 μm filtered SW. Plates were incubated at 18 °C and amoebae subcultured fortnightly by transfer of SW to fresh MYA plates with an additional overlay of 0.2 μm filtered SW as described previously [12]. The isolation of *N. perurans* or related species was confirmed by a species-specific PCR as described below. Cell counting was performed in a haemocytometer and in a TC20 automated cell counter (Bio-Rad, Herts, UK). In order to obtain a clonal culture, an isolated trophozoite was separated and propagated as described above.

Two *N. perurans* clones were used in this study: a “laboratory attenuated” and a “wild type”. The laboratory attenuated clone was passaged *in vitro* 98 times over 3 years. Then its virulence was tested in an *in vivo* challenge as described below. This clone was further cultured and passage 120 was used for the *in vitro* test. The wild type corresponds to a recently isolated clone, which was isolated and passaged 4 times over 60 days before the *in vitro* test.

### 2.3 Species-specific PCR

Three published 18S rRNA gene PCR methods specific for *N. perurans* [31], *N. pemaquidensis* [32] and *N. branchiphila* [33] were used to detect and identify the species of amoebae in the culture.

Genomic DNA was extracted from an aliquot of the established *in vitro* culture, containing 5 × 10^3^ *N. perurans* cells, using the EZ1 DNA tissue Kit and an EZ1 extraction robot (Qiagen, Manchester, UK) following the manufacturer's protocol.

PCR reactions were performed in a 50 μL reaction volume consisting of 1x GoTaq flexi buffer (Promega, UK), 2.5 mM MgCl_2_, 0.25 mM each dNTP, 50 pmol of the forward and reverse primers, 1.25 units of GoTaq^®^ DNA Polymerase (Promega, UK) and 2.5 μL of the extracted DNA. The reaction mix was overlaid with mineral oil and after an initial denaturing step (5 min at 95 °C), was subjected to 35 temperature cycles (1 min at 95 °C, 1 min at 55 °C and 1 min at 72 °C) in a PTC-225 Peltier thermal cycler (MJ Research, Canada) followed by a final extension step of 10 min at 72 °C. PCR products were visualised on 2% agarose gels stained with ethidium bromide and purified using GENECLEAN^®^ (Anachem, UK). Both DNA strands were sequenced using the ABI PRISM™ dye terminator cycle sequencing kit (Perkin Elmer, UK) on an ABI 310 genetic analyser. Sequence similarity searches were conducted using blastn [34] and the NCBI nucleotide database.

### 2.4 Atlantic salmon challenge

An AGD bath challenge was developed using a recently isolated *N. perurans* clone. After 3 years of clonal culture, the virulence of the clone was re-evaluated. In both challenges, a single tank containing 30 Atlantic salmon reared in house, weighing approximately 500 g, were exposed to *N. perurans* by static bath immersion using 5000 trophozoites L^−1^ for 4 h [12]. Then the flow rate was restored to 5–7 L per minute and the water temperature maintained at 16 ± 1 °C. A negative control tank was mock infected. Fish were observed for clinical signs of AGD, which includes rapid opercula movement, gasping at the surface, lethargy and loss of condition. After 4 weeks, fish were humanely terminated by concussion and destruction of the brain, and the gills were examined for scoring the AGD lesions [35], consisting of pale discoloured patches on gills, hyperplasia, and excess mucus production. On the day of termination, the second and the third complete gill arch of one side were dissected and fixed as soon as possible in 10% neutral-buffered formalin (NBF) for 24 h and then following routine histology processing embedded in paraffin wax using a vacuum infiltration processor using standard protocols. Embedded blocks were sectioned at 3–4 μm thickness using a rotary microtome and sections were stained with haematoxylin and eosin (H&E). Sections were examined using a Nikon E800 light microscope with images captured using Lucia™ software. In addition, the second and the third complete gill arch of the other side were fixed in absolute ethanol for DNA extraction. Water samples (20 mL, second challenge only) were taken in triplicate at 4 weeks post challenge, spun at 13000 rpm for 20 min and the pellet resuspended in the digestion buffer for DNA extraction following the manufactures instructions (EZ1 DNA Kit, Qiagen, Manchester, UK). Then presence of *N. perurans* DNA was analysed using the species-specific *N. perurans* PCR as described above.

### 2.5 Inoculation of *N. perurans* on RTgill-W1 cells seeded on Transwell^®^ inserts

**Fig. 1:**
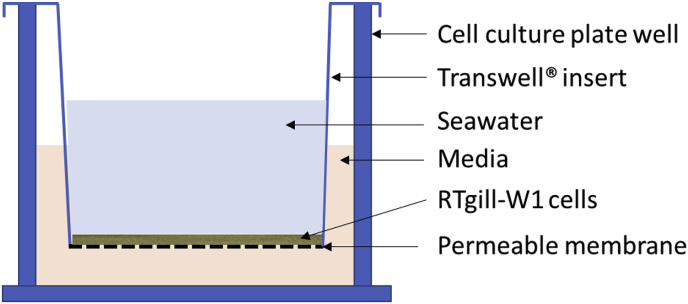
Schematic overview of a Transwell^®^ cell culture insert. Rtgill-W1 cells are seeded onto the polyester membrane. Cell media is added to the lower compartment. Sea water containing Neoparamoeba perurans trophozoites are added to the upper compartment.

For the *in vitro* assay, cultured *N. perurans* were transferred to cell culture flasks in MY broth for one day, allowing the amoebae to attach to the plastic. On the day of the test, the medium was removed, and the amoebae attached to the flask were thoroughly washed three times with sterile SW to remove bacterial contamination. The amoebae were detached from the plastic using 1 mL of 0.05% Trypsin/EDTA Solution Gibco (Paisley, GB), eluted in 10 mL of sterile SW and centrifuged at 500 g for 15 min, the pellet was then resuspended in sterile SW and the number or trophozoites counted as described above. RTgill-W1 cells (ATCC^®^ CRL-2523) were maintained in cell culture flasks in maintenance medium ((MM) L-15 supplemented with 1 mM l-glutamine, 10% fetal bovine serum, Gibco (Paisley, GB) and 1% penicillin-streptomycin (Sigma)) and incubated at 20 °C. Cells were seeded onto Transwell^®^ permeable supports (12 mm inserts, 12 well plate, polyester membrane with 0.4 μm pore size, Corning) each insert containing about 10^4^ cells in a growth area of 1.12 cm^2^. Maintenance media was added to both sides of the membrane, and the cells were incubated at 20 °C for at least 24 h. The day of the inoculation with *N. perurans*, the medium was removed from the chamber above the membrane and replaced with 200 μL of SW containing 4 × 10^3^ trophozoites of either the laboratory attenuated or the wild clone, or just sterile SW as a negative control (schematic in Fig. 1). Inoculated RTgill-W1 cells were incubated at 18 °C and harvested in triplicate at 0, 1, 3, 6, 24, and 48 h post inoculation (pi). For each sampling point, the top SW of each chamber was discarded carefully. Association of the amoebae to the cell monolayer was assumed if the amoebae remained on the monolayer after sea water was discarded. Rainbow trout cells plus the attached amoebae were removed from the filter by addition of 100 μL of trypsin/EDTA (0.05% trypsin, 0.53 mM Na_4_EDTA, Sigma) per chamber.

The cells were then transferred to a 1.5 mL microfuge tube and trypsin inactivated by adding 1 mL of MM. Cells were then pelleted by centrifugation at 500*g* for 5 min and resuspended in 300 μL of RLT lysis buffer (Qiagen, Manchester, UK) for RNA extraction.

In parallel, three Transwell^®^ plates were inoculated to follow the association of amoebae with the RTgill-W1 cells. Cells were monitored at 20 min, and then at 1, 3, 6, 24, 48, 72 and 96 h pi to follow any changes to the cell monolayer including cytopathic effects (CPE). The development of CPEs was examined over the complete culture surface and semi-quantified depending on the area affected: 0 (no CPE), 1 (CPE observed in 25% of cell monolayer), 2 (CPE in 50% monolayer), 3 (CPE in 75%) and 4 (CPE affecting the complete monolayer).

### 2.6 Separation (by filtration) of amoebae, bacteria and extracellular products within the crude *N. perurans* culture

To assess the potential effect of the bacterial component and extracellular products (ECPs) from amoebae and bacteria, the neat *N. perurans* wild type clone culture harvested from the MYA plate was separated by filtration and different fractions were inoculated onto RTgill-W1 cells as described above. Briefly, *N. perurans* trophozoites, of an average size ranging from 15 to 40 μm [36,37], were removed from the culture by filtration using a Minisart filter of 0.7 μm pore size (Sartorius). This first filtration gave a suspension of bacteria and ECP. The suspension was filtered again by using a Minisart filter of 0.2 μm pore size (Sartorius), which enabled the separation (removal) of bacteria of a typical volume 0.4–3 μm^3^ [38,39] from the remaining ECPs derived from the amoebae and co-cultured bacteria. The appropriate filter pore size used to remove bacteria was selected by microscopical observation of the bacteria population (data not shown).

The optical density (OD) of the neat harvest and both filtrates was measured at a wavelength of 600 nm in a WPA Biowave II spectrophotometer (Biochrom). OD values were 0.269 for the crude *N. perurans* harvest; 0.119 for 0.7 μm filtrate containing the bacteria and ECP fractions; and 0.034 for the 0.2 μm filtrate containing only ECPs. The protein concentration in the ECP fraction was further measured using the Bicinchoninic Acid Kit (Sigma) following manufacturer's instructions, giving a concentration of 25 μg/mL.

RTgill-W1 cells on Transwell^®^ were then exposed either to 100 μL of the neat *N. perurans* culture containing approximately 10^3^ trophozoites; 100 μL of the bacteria/ECP suspension; or 100 μL (2.5 μg) of the ECP only fraction. Control cells exposed to SW and to the different treatments were harvested at 0, 3 and 24 h pi as described above.

### 2.7 Phagocytosis assay

pHrodo™ Green conjugated Zymosan Bioparticles™ (Life technologies) were used to visualize trophozoites on the rainbow trout cells.

Briefly, the amoebae were incubated overnight at 18 °C with bioparticles (0.5 mg mL^−1^), and then inoculated onto the RTgill-W1 cells. The pHrodo^®^ Green conjugates are non-fluorescent outside the cell at neutral pH, but fluoresce brightly green at acidic pH, such as when internalised in phagosomes. The nuclei of the cells were counterstained with Hoechst 33342, NucBlue^®^ Live ReadyProbes^®^ Reagent (Invitrogen). Cells and amoebae were viewed with an OLYMPUS^®^ IX83 inverted microscope equipped with a CoolLED Ltd. pE-300 fluorescence illumination unit and OLYMPUS^®^ XC50 camera (Olympus Corporation, Tokyo, Japan). Images were taken at 20x objective magnification under phase contrast and using fluorescence emission wavelengths of 455 nm (DAPI, cell nuclei) and 518 nm (FITC, *N. perurans*) and multi-channel layered images were combined using Olympus cell Sens Dimension 1.15 software.

### 2.8 Scanning electron microscopy (SEM)

SEM was conducted to study the association of *N. perurans* to the *in vitro* gill monolayer. RTgill-W1 cells were seeded onto Transwell^®^ inserts and inoculated either with the *in vitro* cultured amoebae or just SW as negative control as described above. After 4 h, the top SW was removed from the well and the Transwell^®^ inserts washed three times with sterile SW. The cells were fixed by adding 2.5% glutaraldehyde in 0.1 M sodium cacodylate buffer, pH 7.4.

The fixed monolayers were transported to the Animal and Plant Health Agency, Wadebridge, UK for SEM following standard protocols. Briefly, membranes were washed thoroughly in 0.1 M sodium cacodylate buffer and dehydrated in an ascending ethanol series (50–100%), 10 min in each solution. The samples were dehydrated in a BAL-TEC Critical Point-Dryer (CPD 030, Germany), and coated with a thin conductive layer of gold/palladium using a Polaron Sputter Coater (SC7640, UK). The coated samples were mounted on brass stubs, examined and photographed with a Zeiss EVO-50-EP scanning electron microscope in an accelerating voltage of 20 kV in the secondary emission mode.

### 2.9 Taqman qPCR assays

RNA was extracted from each sample using an EZ1 RNA Cell Mini Kit v2.0 and EZ1 extraction robot (Qiagen, Manchester, UK). RNase-free DNase I treatment (Qiagen) was performed during the RNA extraction following manufacture instructions. RNA was diluted in 60 μL of elution buffer. 200 ηg of extracted RNA was reverse transcribed in a 20 μL reaction containing 0.25 mM each dNTP, 500 ng of random primers and 200 units M-MLV reverse transcriptase (Promega, Southampton, UK) at 37 °C for 1 h.

Taqman assays were then performed with 2 μL of cDNA containing 10 ng of input RNA, 500 nM of each primer and 250 nM of probe labelled with 6-FAM in 5′ and MGB in 3’, in a total volume of 20 μL by using the Taqman Universal PCR master Mix with AmpErase UNG (Applied Biosystem). qPCR and fluorescence detection were performed on a StepOne Real-Time PCR, software V2.3 (Applied Biosystem) at 50 °C for 2 min followed by 95 °C for 10 min then 40 cycles of 15 s at 95 °C and 1 min at 60 °C. Each sample was tested in duplicate. Molecular grade water was used as negative control for each master mix.

### 2.10 Gene expression

Twelve rainbow trout genes homologous to Atlantic salmon genes, that through gene expression [40,41] and transcriptome studies [42,43] were observed to be differentially expressed during AGD infection were selected (Table 1). EF-1α was used as a comparator housekeeping gene.

**Table 1:** Summary of the rainbow trout genes and the nucleotide sequences of the primers and probes used for the Taqman qPCR assays: EF-1α, elongation factor EF1 alpha; C3, complement component C3-4; IL1β, interleukin 1 Beta; IL4/13A, interleukin 4/13A; IL6, interleukin-6; IL10, interleukin-10; MHC-1, major histocompatibility complex (MHC) class 1b; iNOS, inducible nitric oxide synthase; lysozyme, lysozyme II; GATA3, GATA binding protein 3; T-bet, tbx21 gene; PCNA, proliferating cell nuclear antigen; AG-2, anterior gradient-2. 500 nM of each primer and 250 nM of probe labelled with 6-FAM in 5′ and MGB in 3’ added in the real-time PCR reaction mixture.

Gene	Accession no.	Source	Forward Primer 5′-3′	Reverse Primer 5′-3′	Probe 6-Fam 5′-MGB 3′
*Reference Gene*					
EF-1α	NM_001124339.1	[76]	TGCCCCTCCAGGATGTCTAC	CACGGCCCACGGGTACTG	AAATCGGCGGTATTGG
*Complement Factors*					
C3	AF271080	[57]	ATTGGCCTGTCCAAAACACA	AGCTTCAGATCAAGGAAGAAGTTC	TGGAATCTGTGTGTCTGAACCCC
*Cytokines*					
IL1β	NM_001124347.2	[77]	CATCACCATGCGCCACATT	GCCACCCTTTAACCTCTCCA	CCAACCTCATCATCG
IL4/13A	AB574337	[57]	ATCCTTCTCCTCTCTGTTGC	GAGTGTGTGTGTATTGTCCTG	CGCACCGGCAGCATAGAAGT
IL6	DQ866150	[57]	ACTCCCCTCTGTCACACACC	GGCAGACAGGTCCTCCACTA	CCACTGTGCTGATAGGGCTGG
IL10	AB118099	[57]	CGACTTTAAATCTCCCATCGAC	GCATTGGACGATCTCTTTCTTC	CATCGGAAACATCTTCCACGAGCT
*Cellular Receptors*					
MHC-1	AY523671.1	[77]	GGAAGAGCACTCTGATGAGGACA	CACCATGACTCCACTGGGG	TCAGTGTCTCTGCTCCA
iNOS	AJ300555.1	[77]	GCCTGCGGTGTCCAACAT	AAAGGGACAGGCTGGAAAT	CTGATGGAGATTGGTGG
*Anti-bacterial Proteins*					
Lysozyme	X59491	[57]	GAAACAGCCTGCCCAACT	GTCCAACACCACACGCTT	ATACCCAGGCCACCAACCGCAACAC
*Transcription Factor*					
GATA3	FM863826	[57]	TCCTGGAGAGAGGGATGAAA	AGCCCGAGACCTATAGCACA	GGCCTTCACTTTCGCCTGCT
T-bet	FM863825	[57]	TTCTGCCATTTTGTGTCAGG	TTCTCCATCCTATTGCTCCAG	TGGTTTTCCTATTGGAAGGCGG
Cell proliferation					
PCNA	KC747822.1	Primers: [43] Probe: this study	TGCCCGTATCTGCCGTGAC	GTGCCCAGCTCTCCCGTG	GCGTCACCAATCTGAGA
AG-2	XM_021560151.1	Forward: [43] Reverse and probe: this study	CCAGTATGTCCCCAGAATCA	CATGTTGCTCAACAAGAGTTG	CGCCTATGAGCCTTC

IL1β expression was measured in cells inoculated with the bacteria and ECP fraction present in the neat *N. perurans* culture in infected and uninfected cells, at 0, 3, and 24 h.

Serial tenfold dilutions of cDNA sample were used to generate standard curves to determine each primer set efficiency, giving slope values close to −3.2. Relative fold changes in gene expression were calculated using the ΔΔCt method (2-ΔΔCt) [44].

### 2.11 Statistical analyses

Gene expression between treatment groups and time points was analysed in two ways. Firstly, average gene expression for each individual gene across treatment groups (wild type and laboratory attenuated clone) was assessed relative to the gene expression observed in control group (sea water) at the corresponding time point. This was done by calculating the mean relative gene expression values (ΔΔCT) and corresponding 95% confidence intervals for each treatment group and testing the significance of the magnitude of observed difference from the control group using REST^©^ [45]. These relationships were visualised through graphical plots generated in R version 3.4.3 [46].

The second analysis focussed on the relationships occurring across the suite of genes studied for each treatment group and time point. To do this pairwise relationships between the normalised (against the house keeping gene) CT values were examined graphically and the level of correlation between results assessed using Pearson's correlation coefficient. A Principal Component Analysis (PCA) was then run on these data, and the results plotted to establish whether any clustering in samples for each treatment group and time point could be observed based on the underlying patterns of gene expression as a whole. These analyses were all conducted in R version 3.4.3 [46].

### 2.12 Absolute quantification of *N. perurans* mRNA

**Fig. 2:**
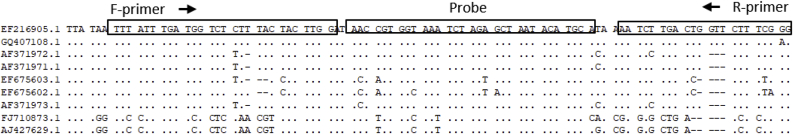
Sequence alignment of the 18S rRNA gene of Neoparamoeba perurans (accession no. EF216905.1 and GQ407108.1), N. pemaquidensis (AF371972.1 and AF371971.1), N. branchiphila (EF675603.1 and EF675602.1), N. aestuarina (AF371973.1), rainbow trout (FJ710873.1) and Atlantic salmon (AJ427629.1). Boxes shown the primers and probe designed for N. perurans Taqman assay.

A specific Taqman qPCR assay to quantify the *N. perurans* mRNA 18S rRNA gene was designed. Specificity of the primers and probe was selected by an alignment with other *Neoparamoebae* species often co-isolated from AGD-affected fish: *N. pemaquidensis* and *N. branchiphila* [see Ref. [34]] and the related species as *N. aestuarina*. Host DNA from Atlantic salmon and rainbow trout was included in the alignment (Fig. 2). *N. perurans* Taqman qPCR primers were designed using Primer Express software (PE Applied Biosystems). The probe was designed by eye to obtain a suitable Tm. Nucleotide sequence and position on the *N. perurans* 18S rRNA gene (Genbank accession no. EF216905.1) is: *N. perurans* Forward: 5′-TTTATTTGATGGTCTCTTTACTACTTGGA-3′, primer position 116–144; Reverse: 5′-CCCGAAAGAACCAGTCAAGATT-3′, position 201-180; and probe: FAM-5′-AACCGTGGTAAATCTAGAGCTAATACATGCA-3′-MGB, position 146–176.

To quantify the number of copies of the *N. perurans* 18S rRNA gene, a fragment of 737 bp, containing the probe region, was amplified using 18S *N. perurans* primers F: 5′-TGTGAAACTGCGAATGGCTC-3′ and *N. perurans* R [13], and cloned into the pGem-T Easy plasmid vector (Promega) and sequenced. The template (dsDNA) copy number was calculated and a plasmid dilution series, from 10^6^ to 1 copy, generated to obtain a standard curve. The regression analysis of the standard curves gave an average slope of −3.5, *p*^2^ 0.99 and a PCR efficiency of 92%.

## 3 Results

### 3.1 Confirmation of the *N. perurans* isolation

Based on a partial sequence of 18S rRNA gene, both clones showed a 100% of identity to *N. perurans* (GenBank accession number EF216905.1). There was no amplification of the 18S rRNA gene for other species from the genus *Neoparamoeba, N. pemaquidensis* and *N. branchiphila*. The species-specific PCR tests were conducted for both clones when the clones were initially isolated, before the challenge to confirm the infectivity of the laboratory attenuated clone and before the inoculation of the cell monolayers (Supplementary S1).

### 3.2 Confirmation of the loss of virulence after long term clonal culture

Atlantic salmon were bath challenged with the laboratory attenuated clone when it was firstly cloned and then after being *in vitro* passaged for 3 years.

In the first challenge, fish showing signs of AGD were sampled at 3 weeks post challenge. Gill gross pathology scored 2 when the animals were sampled. The histopathology confirmed the presence of amoebic cells in the gill lamellae as well as gill hyperplasia. *N. perurans* was confirmed by the species-specific PCR in gill samples.

In the second challenge, there were no signs of AGD in the challenged fish during the four week challenge. When the animals were sampled, there was no gross pathology associated with AGD, giving gill scores of 0. Hyperplasia or other abnormalities were not observed in the histological sections of the gills. *N. perurans* DNA was not detected in the DNA extracted from the gills despite positive detection of one out of the three water samples taken at four weeks post challenge by a single round of PCR.

### 3.3 *N. perurans* association with the gill cell line

**Fig. 3:**
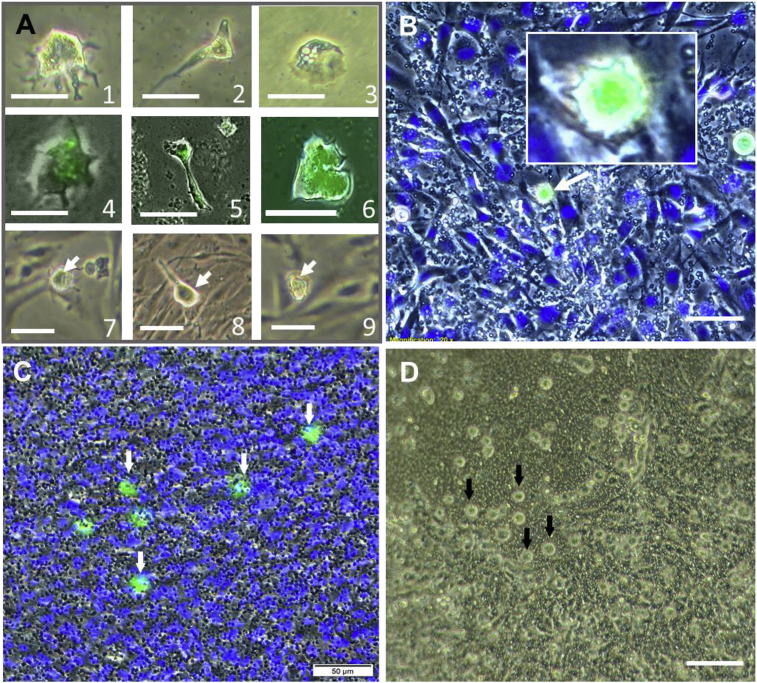
(A) Cultured Neoparamoeba perurans in MYA plates (A1-6) and associated to RTgill-W1 cells (A7-8) showing dactylopodial (A1, 4 and 7), monotactic (A2, 5 and 8) and mamilliform shape (A3, 6, and 9). A4-6: amoeba loaded with Zymosan bioparticles showing bright green fluorescence. (B–D) RTgill-W1 cells seeded on Transwell^®^ inserts. B, C: Cells inoculated with N. perurans (arrows) at 3 h pi. RTgill-W1 cells nuclei counterstained in blue. B top right insert shows detail of a trophozoite loaded with bioparticles seated on the cell monolayer. D: Gill cell monolayer at 96 h pi inoculated with N. perurans (arrows), showing disruption of the cell monolayer. A: bar 25 μm; B, C, D: bar 50 μm. (For interpretation of the references to colour in this figure legend, the reader is referred to the Web version of this article.)

To distinguish amoebae cells from the monolayer cells, *N. perurans* were exposed to zymosan bioparticles. The amoebae successfully incorporated the beads (Fig. 3A), showing phagocytotic capability of the protozoan parasite. The RTgill-W1 cells did not incorporate residual bioparticles.

**Fig. 4:**
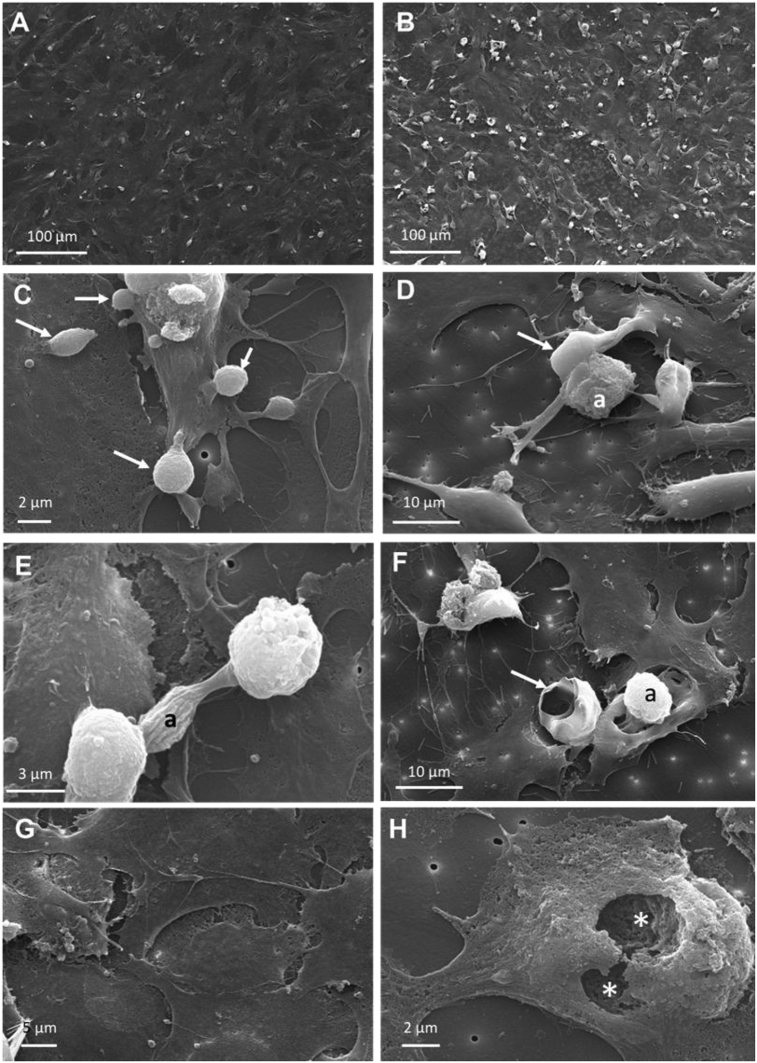
Scanning electron microscopy (SEM) of RTgill-W1 cells seeded on Transwell^®^ insert inoculated with Neoparamoeba perurans at 4 h post inoculation. A, B: SEM micrograph at low magnification, rounded exfoliated cells are observed both in (A) control and (B) inoculated cells. C: Detail of exfoliated cells (arrows) in inoculated monolayer. D: amoeba-like cell (a) showing a malleiform shape and exfoliated cell (arrow). E: amoeba-like cell (a) showing monotactic shape. F: amoeba (a) embedded onto the cell monolayer and exfoliated cell (arrow). G: Detail of control cells at higher magnification. H: Fenestration (asterisks) of the cell monolayer in the inoculated wells.

**Fig. 5:**
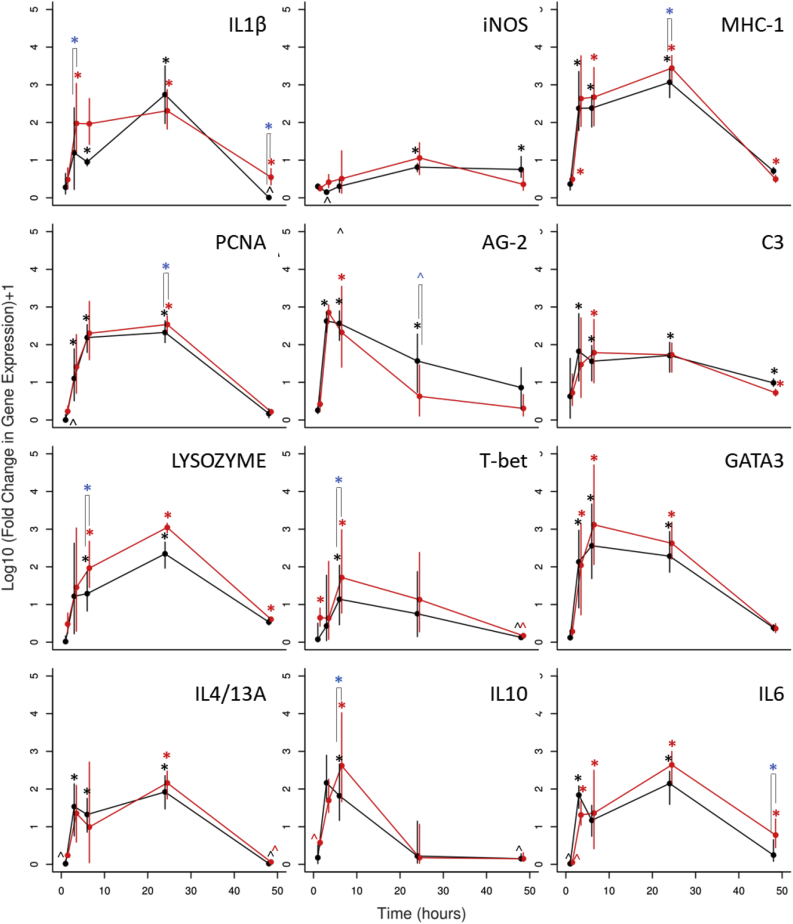
Relative gene expression (fold change) over time (1, 3, 6, 24 and 48 h) of rainbow trout gill cells (RTgill-W1) exposed to a wild type (red) or a laboratory attenuated (black) clone of Neoparamoeba perurans compared to gene expression in seawater exposed cells. Vertical lines represent 95% confidence intervals around the mean. A small offset in time between clones has been applied for visualisation purposes. (*) represents gene expression significantly higher from control group when P ≤ 0.05; (Δ) gene expression significantly lower from control group; and in brackets (⸢⸣) when the gene expression is a significantly higher (blue *) or lower (blue Δ) from cells inoculated with the wild type and compared with the attenuated clone. (For interpretation of the references to colour in this figure legend, the reader is referred to the Web version of this article.)

**Fig. 6:**
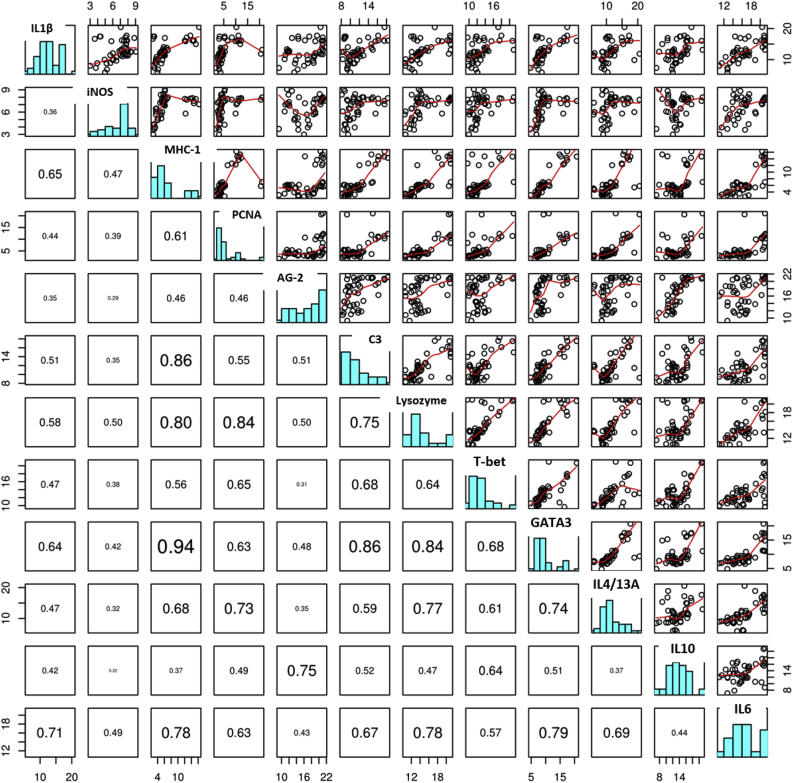
Pairs plots and Pearson's correlation coefficients of gene expression (ɅCT) in Rainbow trout gill cells (RTgill-W1) at different time post exposure to seawater or different isolates of Neoparamoeba perurans.

**Fig. 7:**
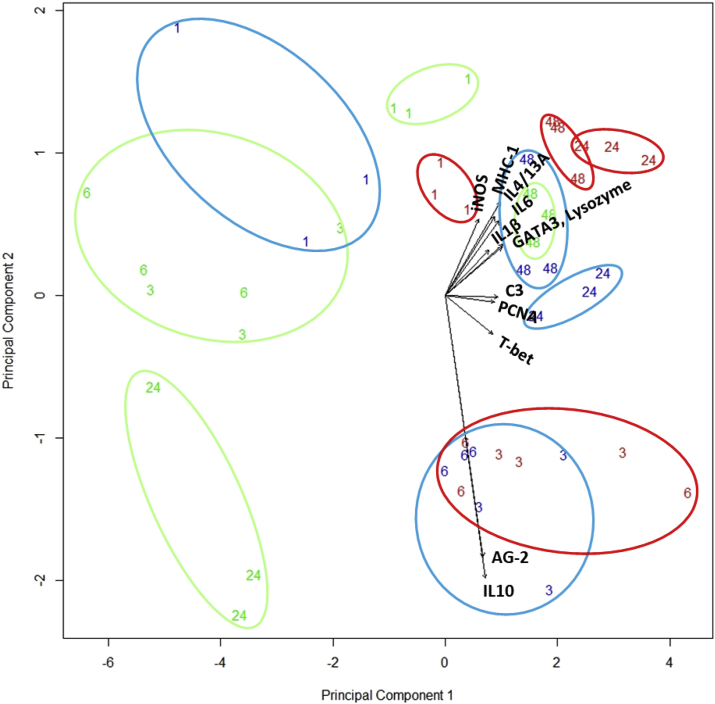
Principal Component Analysis of gene expression of rainbow trout gill cells (RTgill-W1) exposed to seawater (green) or a wild type (red) or a laboratory attenuated clone (blue) of Neoparamoeba perurans. Numbers relate to the hour after exposure that samples were taken. Arrows and labels show the direction of the component loading associated with each gene studied (a ×3 scaling factor has been applied for visualisation purposes). Ellipsoids used to show separations between treatments and time sampling. (For interpretation of the references to colour in this figure legend, the reader is referred to the Web version of this article.)

**Fig. 8:**
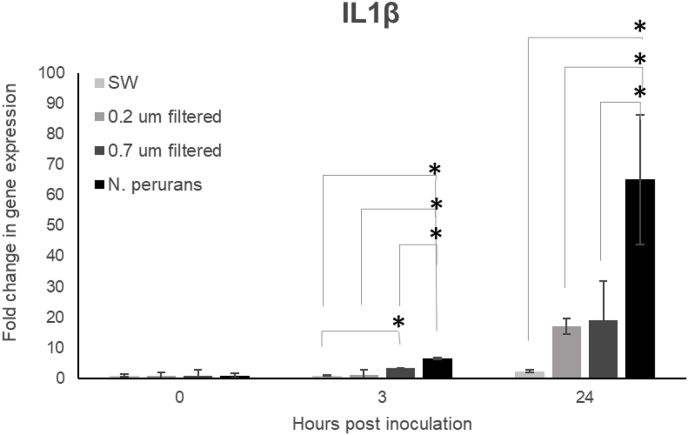
Relative gene expression (fold change) of IL1β over time (0, 3 and 24 h) of rainbow trout gill cells (RTgill-W1) exposed either to the neat Neoparamoeba perurans culture, the bacteria component of the culture (0.7 μm filtrate), or extracellular products (0.2 μm filtrate) compared to gene expression in seawater exposed cells at each time sampling. (*) represents gene expression significantly higher from control group when *P* ≤ 0.05.

When the cells were inoculated with the parasite, firstly amoebae displayed a dactylopodial shape, showing digitiform pseudopodia (Figs. 3A–7), from 20 min to 3 h pi corresponding mostly to floating amoeba. After 3 h however, trophozoites placed on the top of the monolayer showed either a monotactic (Figs. 3A–8) or mamilliform shape (Figs. 3A–9, B-D), with the latter form the most prevalent.

After 6 h pi, free floating amoebae in the top chamber were absent and trophozoites could be observed associated with the cell monolayer (Fig. 3B and C). This pattern was observed for both the attenuated and the wild type clones in cell monolayers inoculated with identical numbers of trophozoites.

Small foci of CPE were observed in the RTgill-W1 monolayer with *N. perurans* from 6 h pi onwards. CPE consisted of vacuolation of cells and lysis with the subsequent loss of cell connection of the monolayer. After 72 h pi, the cell monolayer started to show evident loss of cell confluency (Fig. 3D), compromising epithelial resistance with the diffusion of SW and media through the filter pores. At 96 h pi, the cell monolayer sheet lifted, preventing the continuation of the assay. No CPE were observed in control cells thought out the duration of the test.

SEM observations showed rounded and exfoliated cells in the gill cell monolayer at 4 h pi, both in control and inoculated cells but at a higher proportion in the latter (Fig. 4A–C), which could be due to the cells lifting and detaching from the monolayer. Some amoebae-like cells showed a malleiform shape (Fig. 4D), while other showed monotactic shape (Fig. 4E). In the inoculated cells, amoebae-like cells were observed settled down onto the cell monolayer, however some amoebae-like cells seemed to embed within the cell monolayer (Fig. 4F). Fenestration of the cell monolayer was observed in the inoculated samples. (Fig. 4H).

### 3.4 *N. perurans* growth on the *in vitro* system

The growth of *N. perurans* on the Transwell^®^ plates was quantified by measuring the parasite mRNA associated to the cell monolayer at each sampling point (Table 2). At 1 h pi, the *N. perurans* 18S rRNA mRNA average copy number was 1.3 × 10^6^ for the wild type clone and 3 × 10^6^ for the laboratory attenuated clone. The highest number of copies was measured at 3 h pi (1.4 × 10^7^) for the attenuated clone and at 1 h for the wild type. Then, a general decrease in the number of copies was seen at every sampling point for both clones, ranging from 10^6^ copies at 1 h to 10^3^ copies at 72 h pi. At 96 h, the cell monolayer was mostly disrupted and lifted, and it was not possible to detect the amoebae RNA in the remaining cell monolayer. The amoeba 18S rRNA gene was not detected in control cells at any time sampling.

**Table 2:** Quantification of the *Neoparamoeba perurans* mRNA 18S rRNA gene. For each sample, 2 μL of cDNA containing 10 ηg of input RNA were analysed. Number of replicates at each sampling time = 3. Wild type refers to the wild type clone; attenuated refers to the avirulent cloned attenuated through long term *in vitro* clonal culture.

Hours pi	Ct values (average ± sd)		No. of transcripts 18S rRNA gene	
	Wild	Attenuated	Wild	Attenuated
1	20.4 ± 0.0	19.5 ± 0.7	1.3 × 10^6^	3.0 × 10^6^
3	21.2 ± 3.2	17.6 ± 0.8	7.2 × 10^5^	1.4 × 10^7^
6	21.0 ± 2.0	20.1 ± 0.5	8.5 × 10^5^	1.7 × 10^6^
24	26.7 ± 2.0	21.9 ± 1.5	7.9 × 10^4^	4.0 × 10^5^
48	25.5 ± 2.1	25.4 ± 06	2.0 × 10^4^	2.3 × 10^4^
72	28.7 ± 3.7	28.8 ± 0.0	1.5 × 10^3^	1.3 × 10^3^
96	Undetected	Undetected	–	–

### 3.5 *In vitro* study of host innate immune response to *N. perurans*

Both *N. perurans* clones induced a similar pattern of differential gene expression in the gill cell monolayer compared with control cells (Fig. 5, Supplement 1). The rainbow trout anterior gradient (AG)-2 gene was the earliest responder, with a peak at 3 h pi, followed by the transcription factors T-bet and GATA binding protein 3 (GATA3) with the highest gene expression measured at 6 h pi. The cytokine interleukin 10 (IL10) showed the greatest gene expression at 6 h pi, returning to basal levels (gene expression similar to the sea water control) at 24 h pi. The gene expression of the cytokines IL4/13A, IL6 and IL1β were maintained significantly higher than control cells for at least 24 h pi. Similar pattern was observed for the major histocompatibility complex class 1 (MHC-1), proliferating cell nuclear antigen (PCNA), lysozyme and complement C3 with the greatest up-regulation in the gene expression at 24 h pi. The gene expression of the inducible nitric oxide synthase (iNOS) was only different from control cells at 24 and 48 h pi for the attenuated clone.

In general, the wild type clone induced a greater up-regulation (*p* < 0.05) than the attenuated clone of IL1β at 3 and 48 h; lysozyme, T-bet and IL10 at 6 h pi; MHC-1 and PCNA at 24 h; and IL6 at 48 h. As the exception to this, the attenuated clone induced significantly more AG-2 gene expression at 24 h.

Fig. 6 shows associations between the level of gene expression in each of the genes studied. In most cases genes responded in a similar way, showing relatively high levels of positive correlation. In particular, MHC1 was very strongly correlated with C3, Lysozyme and GATA3, each of which was in turn strongly correlated to one another and may therefore suggest MHC1 could be a good surrogate for these genes in future studies. IL1B, IL10, iNOS and AG2 were the exception to this rule, showing little association with each other and appearing to operate independently to each of the other genes studied.

Principal components 1 and 2 (Table 3, Fig. 7) explained 72% of the variability in the dataset. Component 1 explains 62% of the variability in the gene expression dataset and is associated with all the genes responding in the same way as each other, as suggested by the results of correlation analysis described above (Fig. 6). However, the four genes showing the least associations with the other genes in the pairwise analysis demonstrated the lowest contribution to this component (IL1B = −0.26, iNOS = −0.20, AG-2 = −0.22 and IL10 = 0.24). The genes found to be most strongly correlated in the pairwise analysis; C3, Lysozyme and GATA3 were also found to contribute the most to this component.

**Table 3:** Principal component loadings relating to gene expression in rainbow trout gill cells (RTgill-W1) at different time post exposure to seawater or different isolates of *Neoparamoeba perurans*. Numbers in parenthesis relate to the proportion of the variability in the dataset explained by each loading.

	PC1 (0.62)	PC2 (0.10)
IL1B	−0.26	−0.11
iNOS	−0.20	−0.18
MHC-1	−0.33	−0.22
PCNA	−0.29	0.01
AG-2	−0.22	0.61
C3	−0.31	0.00
Lysozyme	−0.34	−0.11
TBET	−0.28	0.09
GATA3	−0.34	−0.12
IL4-/13A	−0.29	−0.18
IL10	−0.24	0.66
IL6	−0.32	−0.17

Component 2 explains a further 10% of the variability in the dataset, and is largely explained by changes in two genes, IL10 and AG2. The loadings for these genes are positive and considerably higher than for all other genes, which are generally negative and contribute little to the component score. This suggests that under some of the circumstances investigated in this study they are expressed independently of the other genes.

Plotting each component score for all the samples shows clear clustering between time points and between treatment groups and controls. Control cells showed different gene expression over time. However, cells inoculated with both clones showed a clear separation from the control cells at the points 3 and 6 h pi, with high expression of AG-2 and IL10. By 24 h all of the exposed samples were showing high expression of all genes compared to 24 h seawater controls. Finally, by the 48 h sample point, all samples, treated and untreated, clustered closely, showing a similar pattern of gene expression.

### 3.6 RTgill-W1 inflammatory response to bacteria and ECP components of the neat *N. perurans* culture

Cells inoculated with the neat *N. perurans* culture showed a significant up-regulation of IL1β at 3 and 24 h pi compared with both control cells and cells inoculated with either the bacteria fraction and/or ECPs. Cells inoculated with the bacteria filtrate showed a significant up-regulation of IL1β at 3 h pi but not at 24 h pi probably explained by the increase in the IL1β expression of control cells and high variability between replicates on the inoculated cells at this time point. Finally, cells inoculated with the ECP filtrate did not show a significant up-regulation of IL1β at the concentration tested (Fig. 8).

## 4 Discussion

The aim of this study was to test an *in vitro* platform as a proxy for investigating *N. perurans*-host interaction. For this purpose, the observed gene expression of immune-associated genes in this *in vitro* platform was then compared with the published *in vivo* host response to *N. perurans* (Table 4).

**Table 4:**
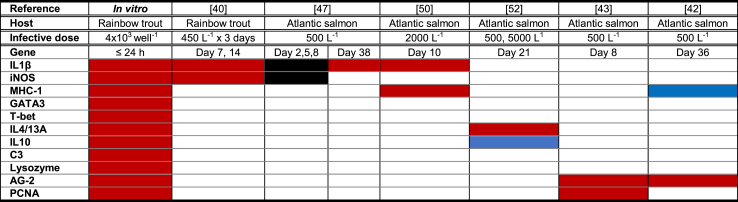
Comparison of the rainbow trout gene expression in the *in vitro* model (RTgill-W1 cells seeded on Transwell^®^ inserts) with the gene expression of salmonid gills infected with *Neoparamoeba perurans*. Red shows up-regulation, blue shows down-regulation and black shows no change to the control group. Infective dose: number of trophozoites.

In salmonids, *N. perurans* infection triggers a significant up-regulation of the pro-inflammatory cytokine IL1β in the gills of infected rainbow trout [40] and Atlantic salmon [41,47]. In the present *in vitro* platform, IL1β gene expression was significantly up-regulated after 3 h of exposure to *N. perurans* and kept over expressing even in the absence of *N. perurans* growth, with a peak at 24 h for both clones. In AGD-affected Atlantic salmon tissue, a chronic IL1β over expression has been observed despite the AGD lesions showing very little evidence of inflammation [48].

The production of nitrogen radicals is an antimicrobial mechanism which has been previously reported in gills of rainbow trout [49]. In the present *in vitro* system a moderate up-regulation iNOS was measured. Up-regulation of the cellular receptor iNOS in infected rainbow trout but not in Atlantic salmon has been reported in AGD lesions showing characteristic AGD histopathology [40,47].

In the *in vitro* model, MHC-1 was highly up-regulated from 3 to 24 h. In Atlantic salmon, MHC-1 mRNA was observed up-regulated in AGD-lesions at 10 days post infection (pi), suggesting a classical inflammatory response in the gills of AGD-affected fish [50]. However, down-regulation of the antigen presentation pathway transcripts in Atlantic salmon has been reported at 36 days post infection in gills showing focal AGD-lesions [27,42]. Interestingly, gill transcriptome studies have shown differences in host susceptibility to AGD. Resistant individuals displayed higher expression of genes involved in adaptive immunity and negative regulation of the cell cycle. While, AGD-susceptible individuals, showed higher expression of acute phase proteins and positive regulators of the cell cycle [51]. A modification of the proposed *in vitro* model could be used to cost-effectively screen family susceptibility to *N. perurans* in AGD resistant breeding selection, using primary gill derived cell cultures from different families.

The polarization of the T helper type 2 (Th2) subset has been reported *in vivo* at 21 days pi [52]. *In vitro* models using epithelial cells with parasitic helminth products have shown that up regulation of GATA3 is essential for a Th2 in *vitro* differentiation of T cells [53,54]. In concordance with those results, an early up regulation of the transcription factors GATA3 and T-bet were measured in the inoculated gill cell line. In the present *in vitro* model, the pivotal Th2 cytokine IL4/13A and the effector IL6 mRNA expression were up-regulated as has been reported in gills of AGD positive Atlantic salmon [52]. The Th2 promotor and effector cytokine IL10 also showed a marked-up regulation at 6 h pi in the *in vitro* model. In *in vivo* studies, a down-regulation of IL10 was measured at 21 days pi. Allergic mechanisms caused by *N. perurans* has been suggested for AGD infected fish [52]. The ease of undertaking time series sampling in this *in vitro* model allows for further comprehensive studies of Th2 response to this parasite.

In the *in vitro* model, the gene expression of the complement factor C3 was up-regulated from 3 h onwards. Premature C3 up-regulation has been found in transcriptome analysis of AGD positive Atlantic salmon sampled just after the parasite infection [43]. C3, coupled with expression of IL4, plays a critical role in both Th1 and Th2 responses to an antigen [55].

Lysozymes are glycoside hydrolases which damage bacterial cell walls [56]. In rainbow trout, the ectoparasite *Ichthyobodo necator* triggers an increase in lysozyme in the skin of infected fish suggesting a role for lysozyme in the clearance of extracellular parasites as well as bacteria [57]. In the present *in vitro* model, lysozyme was also found up-regulated. Serum lysozyme levels were unaffected in AGD infected Atlantic salmon [58,59]. While serum lysozyme activity was unchanged, mucus lysozyme activity was significantly increased over time in Atlantic salmon exposed to *N. perurans* on a commercial farm [60] and in an experimental AGD infection [27].

Transcriptome analyses have revealed that an Atlantic salmon homologue of the Xenopus AG-2 (XAG-2) gene was consistently up-regulated in AGD lesions [43]. Histochemistry examination of AGD-positive gills confirmed abundant expression of AG-2 in mucous cells of infected gill [61]. In the RTgill-W1 cells, the gene expression of the rainbow trout AG-2 was significantly up-regulated. RTgill-W1 cells are believed to have derived from undifferentiated precursor gill stem cells. Given appropriate conditions, mucus-secreting goblet-like cells and cells with abundant mitochondria have been reported in this gill cell line [29]. Furthermore, the gene expression of PCNA was also significantly up regulated in the inoculated cells. PCNA is a P53 induced protein [62] with function in cell cycle regulation and DNA replication [63] and as a result it is used as an indicator of cell division and proliferation. In AGD lesions, PCNA was significantly up-regulated relative to samples from healthy fish [43].

The PCA test has shown that AG-2 and IL10 are key indicator genes involved in the host response to *N. perurans*, which can allow for a reduction in the number of host genes analysed when analysing multiple samples [64].

Thus this *in vitro* model has shown a host response to the parasite *N. perurans* which mimics that described *in vivo*. Both *N. perurans* clones tested in the artificial gill epithelium trigged a similar pattern of up regulation of the selected host genes. However, the wild clone triggered a greater gene expression of IL1β, MHC-1, PCNA, lysozyme, T-bet, IL6, IL10 and AG-2 compared to the laboratory attenuated clone for at least some exposure times. In the present study, the loss of virulence of the long term cultured *N. perurans* clone, 98 times passaged *in vitro*, was proved by the lack of AGD lesions in the challenged Atlantic salmon as reported for other avirulent *N. perurans* isolates [23]. The virulence of the wild type clone, *in vitro* cultured for 60 days, was not tested *in vivo* at the time of the *in vitro* test. A clone of *N. perurans* 70 days in culture was able to induce AGD in challenged Atlantic salmon [12]. Whether the host response in this *in vitro* model can confidently be used to discriminate between virulent and avirulent clones requires further investigation using a greater number of isolates, dose-response studies and appropriate controls. Furthermore, this is the first study in showing a host response to an avirulent *N. perurans* clone, which can be used to study pathogenesis and vaccine development.

Despite the successful *in vitro* replication of the host innate response to the parasite there are some aspects of this *in vitro* platform which need further development. In the present study, this model addressed the main limitation of *in vitro* studies which is a requirement for an experimental system which is optimal for both host cells and *N. perurans* [see 28]. The RTgill-W1 cell line was tested as a host cell model. RTgill-W1 cells have shown tolerant growth on Transwell^®^ inserts after an osmolality change from 330 mOsm kg to 1 of the culture medium to 1000 mOsm kg-1 of the artificial sea water, caused by the replacement of the top media by SW [29]. Time-course changes in the gene expression of control cells exposed to SW are shown in the PCA graph which reflects the dynamics of biological processes of adaptation to the new media [65]. Gene expression of infected cells was therefore compared with the respective control samples at each time point. A refinement to explore in future studies could be *ex-vivo* gills or salmon derived primary gill culture onto Transwell^®^ inserts using a novel double-seeded technique, where a tight epithelium is formed with a pavement cell/mitochondria-rich cell ratio similar to that observed *in vivo* [28,66]. Similarly, the *in vitro* model cell culture conditions may be improved by use of homologous supplemental sera from trout rather than bovine origin or increased membrane surface area [67].

The second challenge to develop an *in vitro* culture system is to assure optimal growth conditions for the parasite. *N. pemaquidensis*, often co-isolated from AGD lesions [13], showed rapid growth in RTgill-W1 cells with high osmolarity media (above 700 mOsm Kg-1), causing focal lesions in the cell monolayer within 24 h of exposure, and total destruction of the cell monolayer within 48–72 h pi [26]. Equally, other amoeba species, i.e. *Acanthamoeba* spp. and *Naegleria* spp. have shown to induce cell cytolysis as soon as 48 h pi [68,69]. In the present study, *N. perurans* did not grow in the conditions tested, showing a decrease in the 18S rRNA mRNA of 3 logs after 72 h. Despite the lack of proliferation of *N. perurans* in the Transwell^®^ plate, the association of the amoebae to the gill epithelium and the generation of CPE in the inoculated cells were similar as that described for *N. perurans* [23]. In the present *in vitro* model, due the cytolysis of the cell monolayer induced by the amoeba, control cells did not show CPEs, 72 h is the maximum time that the test can be used in its current form. A solution to explore in future studies would be re-seeding the membrane with cells every 24 h to avoid changes in the osmolarity, this approach would allow amoeba proliferation and longer time-scale studies. Early changes in the osmolarity can be monitored by transepithelial electrical resistance analysis [70].

In our study there was no difference in the induction of CPE on the inoculated cells between the three year old attenuated clone and the wild one. Lack of CPE on Chinook salmon embryo cells has been reported for an avirulent long term cultured *N. perurans* [23]. The loss of virulence of *N. perurans* has been suggested to be due lack of attachment to the gills [23]. In the tested *in vitro* platform, the amoebae were confined in a small volume with contact to the cell monolayer. The association of the avirulent *N. perurans* with the cell monolayer onto the Transwell^®^ plate will be re-evaluated in future studies using cell culture systems with flow through the apical side of the Transwell^®^ support [71]. The loss of virulence of *N. perurans* has also been suggested to be due to the absence of an extracellular product (ECP) [23]. Secreted cytopathogenic compounds have been reported in other amoeba species [72,73]. For *Acanthamoeba* spp. and *Naegleria* spp the pattern of CPE has been used to discriminate between pathogenic and non-pathogenic amoeba species and strains, but often the reproducibility of CPE assays was low [68]. Although there was no significant up-regulation of IL1β associated to the ECP component of the culture, the pattern of increased expression suggests that this *in vitro* platform could be a powerful tool to compare the host inflammatory response to ECP derived from the attenuated and wild clones in future studies by refined time-dose assays. *N. perurans* is currently co-cultured with a bacteria population derived from the AGD infected tissues. Efforts to obtain an axenic culture have failed due to antibiotic resistance of the bacteria and a decreased growth rate of the amoebae exposed for long time to antibiotics (personal comm, data not shown). It is unknown whether *N. perurans* requires live bacteria to survive. Other free living amoebae, such as Naegleria and Acanthamoeba, can be both cultured axenically in cell-free media or on tissue culture cells and in cultures with bacteria as a food source [74]. We have shown that in the absence of an axenic culture, each component of the culture (trophozoites, bacteria and ECPs) can be separated by filtration, and the inflammatory host response to each component can be discriminated in the *in vitro* model. In the present study, the phagocytic activity of *N. perurans* has been proven by the incorporation of Zymosan bioparticles. It has been shown that the phagocytic activity of the parasitic protozoan *Entamoeba histolytica* correlates with its virulence *in vivo* [75]. It is unknown if the same relationship of phagocytosis activity-*in vivo* virulence applies for the *N. perurans*, which can be a subject of future studies using the protocol described in this manuscript.

Finally, SEM analysis identified similar features between the amoeba attachment in infected gills of Atlantic salmon and the association of the parasite to the gill epithelia *in vitro*. In the artificial gill epithelium, the amoebae cells were embedded within the epithelium inducing erosion of the cell monolayer. In fish, the amoeba-epithelial cell interaction resulted in fenestrated indentations of the gill epithelium corresponding to the presence of pseudopodia [36].

## 5 Conclusion

An *in vitro* model using a derived rainbow trout gill epithelium seeded on Transwell^®^ inserts has been shown to be a promising tool to study host cell-amoebae association and host response to *N. perurans*. The Th2 subset and key genes involved in cell proliferation identified in *in vivo* studies were significantly up-regulated in the inoculated cells. This *in vitro* platform could be a valuable tool to test disinfectant and therapeutic compounds to treat AGD and has a potential to test host resistance to AGD in selective breeding programmes.

## Author contributions

RP, IC and BN conceived and designed the experiments; IC performed the experiments; IC and NT analysed the data; AB, SG isolated and maintained the parasite culture; RM performed the live imaging assays; KB assisted with the SEM; IC, NT, RP and BN drafted and revised the manuscript. All authors acknowledged the manuscript.

## Conflicts of interest

The authors declare that the research was conducted in the absence of any commercial or financial relationships that could be construed as a potential conflict of interest.

## Footnotes

Appendix A Supplementary data to this article can be found online at https://doi.org/10.1016/j.fsi.2018.11.029.
