# Supplementary material for: In vitro gill cell monolayer successfully reproduces in vivo Atlantic salmon host responses to Neoparamoeba perurans infection
Source: Fish Shellfish Immunol. 2019 Mar;86:287–300. doi: 10.1016/j.fsi.2018.11.029 (PMC6380893; doi:10.1016/j.fsi.2018.11.029)

**Supplementary data**

**S1.** Agarose gel (2%) showing species-specific PCR test. Expected band size: *N. perurans* PCR: 634 bp; *N. branchiphila*: 1250 bp; *N. pemaquidensis*: 609 bp. Band amplified from the AGD +ve fish sequenced to confirm *N. perurans*.


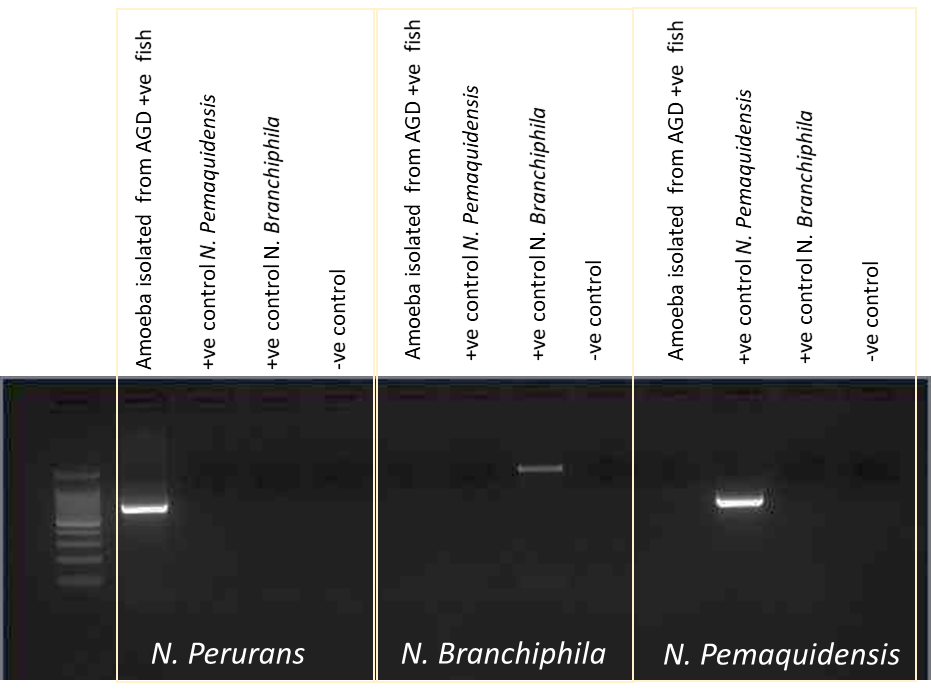

Supplement: Supplementary data [file mmc1.docx]
